# Intracellular G-actin targeting of peripheral sensory neurons by the multifunctional engineered protein C2C confers relief from inflammatory pain

**DOI:** 10.1038/s41598-020-69612-9

**Published:** 2020-07-30

**Authors:** Derek Allen, You Zhou, Audrey Wilhelm, Paul Blum

**Affiliations:** 1 School of Biological Sciences, University of Nebraska, E234 Beadle Center, Lincoln, NE 68588 USA; 2 Center for Biotechnology, University of Nebraska, E234 Beadle Center, Lincoln, NE 68588 USA

**Keywords:** Cell biology, Neuroscience, Biologics, Pain

## Abstract

The engineered multifunctional protein C2C was tested for control of sensory neuron activity by targeted G-actin modification. C2C consists of the heptameric oligomer, C2II-CI, and the monomeric ribosylase, C2I. C2C treatment of sensory neurons and SH-SY5Y cells in vitro remodeled actin and reduced calcium influx in a reversible manner. C2C prepared using fluorescently labeled C2I showed selective in vitro C2I delivery to primary sensory neurons but not motor neurons. Delivery was dependent on presence of both C2C subunits and blocked by receptor competition. Immunohistochemistry of mice treated subcutaneously with C2C showed colocalization of subunit C2I with CGRP-positive sensory neurons and fibers but not with ChAT-positive motor neurons and fibers. The significance of sensory neuron targeting was pursued subsequently by testing C2C activity in the formalin inflammatory mouse pain model. Subcutaneous C2C administration reduced pain-like behaviors by 90% relative to untreated controls 6 h post treatment and similarly to the opioid buprenorphene. C2C effects were dose dependent, equally potent in female and male animals and did not change gross motor function. One dose was effective in 2 h and lasted 1 week. Administration of C2I without C2II-CI did not reduce pain-like behavior indicating its intracellular delivery was required for behavioral effect.

## Introduction

Acute peripheral pain can be treated with anesthetics which inhibit ion channels^1^ and thereby formation or propagation of an action potential required for neuronal signaling. For example, lidocaine (xylocaine)^2,3^ targets sodium channels NaV1.3^4^ and NaV 1.7-NaV1.9^4,5^ that are implicated in pain. Calcium channels play an additional role in pain perception because action potentials promote membrane depolarization leading to calcium influx^6^. In neurons, calcium channel depolarization opens other channels, releases neurotransmitters^7^^,^ and acts as a second messenger in neuronal signaling^8^. These channels include high and low voltage activated families. High voltage families consist of five different subclasses of channels: L, N, P, Q, and R. The N-type are found throughout the body and play a major role in neurotransmitter release in neuronal cells^9^. They transmit pain signals from peripheral neurons through secondary neurons to the central nervous system. For this reason calcium channels are a target for pain therapeutics^10^.

Neuronal signaling also depends on the cytoskeletal protein actin^11^. Actin occurs as monomeric subunits (G-actin), or in polymerized form (F-actin) in the neuron cell body, axon and dendrite. Treadmilling is the reversible process that maintains and remodels F-actin through addition and removal of G-actin^12^. Inhibition of this process using actin inhibitors can modulate formation of an action potential through reduction of calcium influx^13^. In addition, the modulation of actin polymerization has been shown to inhibit sodium influx^14^. Inhibition of an action potential offers a route for pain reduction because it can block peripheral neuronal signaling^15^. However, all known actin inhibitors are small untargeted and membrane soluble molecules that lack cell type specificity^16,17^. Clinical use was abandoned because of reliance on excessive dosage to overcome untargeted diffusion thereby promoting irreversible inhibition of actin polymerization^18^.

C2C is a recombinant protein-based actin inhibitor^19^. It was derived from the *C. botulinum* serotype C toxin called C2 with a retargeted cell specificity through C-terminal replacement of its native binding domain with that of another *C. botulinum* serotype C protein called C1^19^. This conferred on the modified protein the ability to bind the GT1b subclass of gangliosides^20^ that occur on animal neurons^21^ in a protein receptor-independent manner unlike Botox^22^. The native form of C2 is composed of two non-covalently associated proteins called C2I and C2II. C2I is a G-actin ADP-ribosyltransferase that inhibits F-actin formation by blocking polymerization. C2II is a heptameric oligomer that binds target cells and translocates C2I into the cytoplasm. C2II with its engineered C-terminus called C2II-C1, spontaneously oligomerizes, associates with C2I and then binds cells in a GT1b-dependent manner^19^. The C2I/C2II-C1 complex is then internalized by clathrin and Rho-dependent mechanisms within endosomes. Acidification of the endosome causes membrane pore formation by C2II-C1 oligomers followed by C2I dissociation and diffusion into the cytoplasm. Interestingly, unlike other actin inhibitors, C2I controls actin remodeling effects without killing mammalian neurons (no activation of apoptosis)^23^. In addition, and unlike C1, C2 is not a neurotoxin and is not associated with botulism therefore C2C is not a toxin. Here the effects of C2C on neurons was studied in vitro and in vivo. Its apparent effects on calcium channels and specificity towards neuronal subclass, prompted an assessment of its effects on pain-like behaviors using a conventional animal pain model.

## Results

### Inhibition of actin polymerization

**Figure 1:**
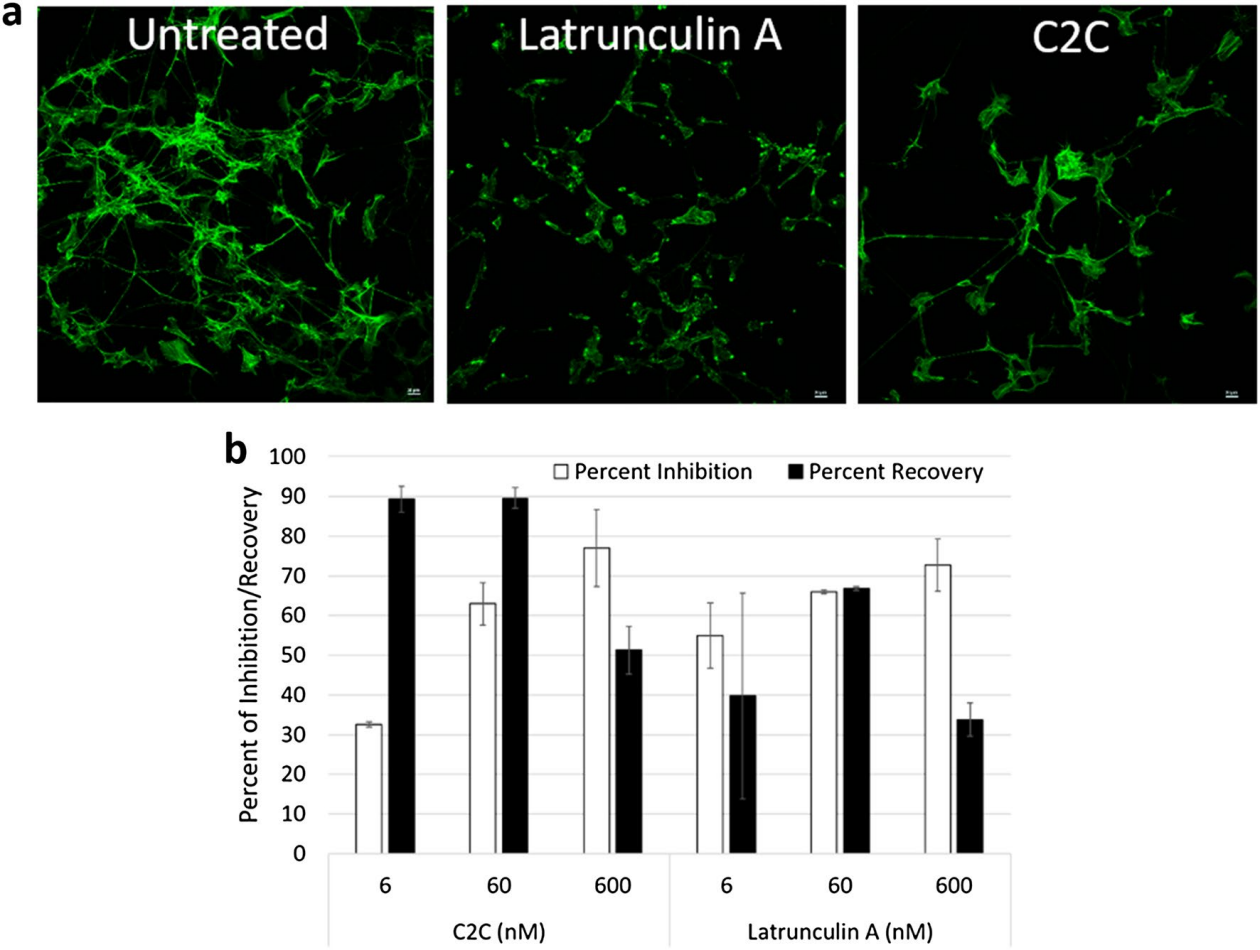
Inhibition and reversibility of actin polymerization. Polymerized F-actin was measured using an alexa fluor conjugated phalloidin stain and imaged by confocal microscopy or quantitated using a microtiter plate reader. The effect of C2C treatment compared to latrunculin A was tested using primary chicken sensory neurons using fluorescent confocal microscopy (**a**). The effect of C2C treatment relative to latrunculin A was quantitated for the percentage of F-actin inhibition and reversibility using GT1b-positive SH-SY5Y cells (**b**). Error bars represent the standard deviation between replicates.

The native C2 subunit C2I inhibits F-actin polymerization through ADP-ribosylation of G-actin leading to actin remodeling^24^. To confirm the occurrence of similar activity by C2C, its effect on actin polymerization was tested. Primary chicken sensory neurons were cultured as described previously^25^^,^ followed by treatment with 60 nM C2C or latrunculin A and staining of treated cells with the F-actin stain phalloidin^26^. Microscopic analysis showed a reduction in F-actin staining for both C2C and latrunculin A treated cells compared to an untreated control cell line though the effect of latrunculin A appeared more significant at this dose (Fig. 1a). To quantitate the effect of C2C treatment and better compare its effects to latrunculin A, the assay was repeated using SH-SY5Y human neuroblastoma cells (Fig. 1b). SH-SY5Y cells were treated with a range of doses of C2C and latrunculin A from 6 to 600 nM for 2 h, followed by staining with phalloidin. This resulted in a dose dependent inhibition of polymerized actin by both agents. As small molecule actin inhibitors like latrunculins have been described as irreversible^27^^,^ the reversibility of inhibition of actin polymerization was tested. SH-SY5Y cells were treated with C2C or latrunculin for 2 h, followed by treatment removal, media replacement and a recovery period of 48 h (Fig. 1b). The effect of C2C treatment was more reversible than latrunculin at particular doses. While 88% and 89% respectively of actin polymerization was observed using C2C doses of 6 and 60 nM, only 66% and 40% respectively was observed with latrunculin A at identical doses. This suggests the effect of C2C was more reversible than latrunculin A.

### Inhibition and reversibility of calcium influx

**Figure 2:**
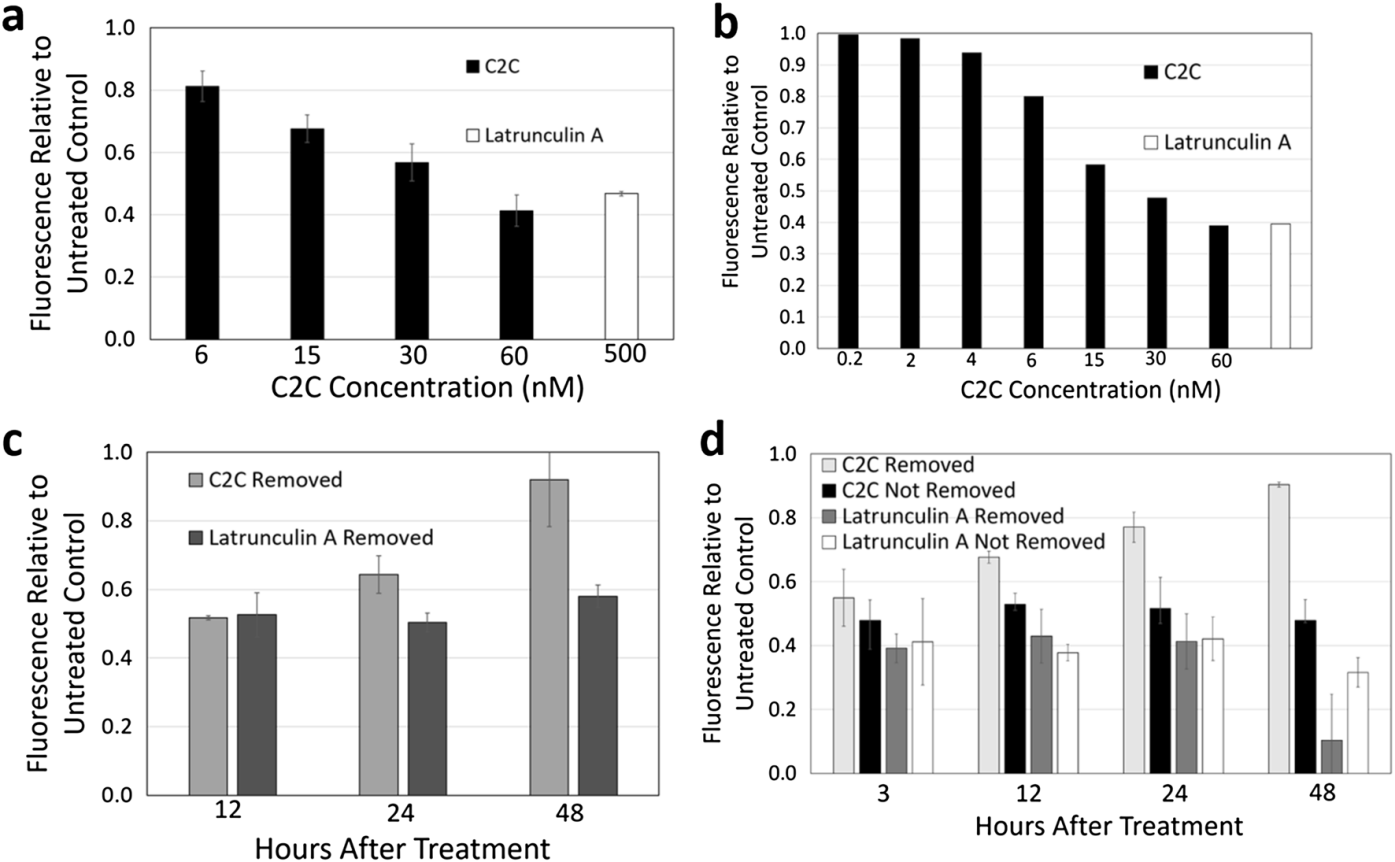
Inhibition and reversibility of calcium influx. Calcium influx was measured using Fluo 4 calcium indicator and a fluorescent microtiter plate reader. The effect of C2C treatment relative to latrunculin A was examined using primary chicken sensory neurons (**a**). The effect of C2C treatment relative to latrunculin A was tested in GT1b-positive SH-SY5Y cells (**b**). The effect of removal of C2C and latrunculin was also examined with primary chicken sensory neurons (**c**) along with the effect of removal from SH-SY5Y cells (**d**). Error bars represent the standard deviation between replicates.

Neuronal influx of calcium ions is required to create or propagate an action potential for signaling^28^. C2II-C1 delivers the G-actin ribosylase, C2I, to the cytoplasm^19^ where it inhibits F-actin formation through post translational modification of G-actin^29^. Because depolymerization of F-actin mediated by latrunculin A has been shown to disrupt calcium influx^30^^,^ the ability of C2C to inhibit calcium influx was examined. Primary chicken sensory cells prepared as described^25^ were treated with C2C and then with the calcium fluorescent dye, Fluo-4^31^. C2C treatment reduced calcium influx as indicated by a reduction in fluorescence at a range of concentrations of C2C (Fig. 2a). To expand on these results using a more rapid and quantitative test, the effect of C2C on calcium influx was examined using the GT1b-positive human neuroblastoma cells, SH-SY5Y^32^ (Fig. 2b). SH-SY5Y cells were plated on black sided 96 well plates, then treated with C2C at doses ranging from 0.2 to 60 nM, or latrunculin A, at a dose of 500 nM. Calcium influx was again reduced by C2C treatment exhibiting a maximum effect of 61% reduction of intracellular calcium compared to an untreated control at 60 nM C2C. The minimum effective dose that reduced calcium influx was 0.2 nM C2C with a 2% reduction compared to an untreated control. The effect of C2C treatment at different doses were sigmoidal in pattern indicating saturation of the GT1b receptor and or the G-actin target. However, neither C2C or latrunculin A were fully effective at blocking calcium influx indicating the activity of other nonresponsive ion flux channels in this cell line^33^. In addition, sodium influx was measured using SBFI sodium indicator and a fluorescent microtiter plate reader with SH-SY5Y cells (Fig S1). SH-SY5Y cells were treated with the indicated doses of C2C for 2 h prior to analysis. No significant reduction in sodium influx was apparent with C2C while latrunculin A elicited a 40% reduction.

Because small molecule actin depolymerizing agents have been shown to have an irreversible effect on calcium influx^27,34^ the reversibility of C2C treatment on calcium influx was examined. Primary chicken sensory neurons were treated with C2C or latrunculin A for 2 h followed by their removal (Fig. 2c). Removal of C2C led to the gradual recovery of calcium influx reaching a capacity of 95% after 48 h after C2C removal. SH-SY5Y cells were treated with C2C or latrunculin A for 2 h followed by either their removal or in separate tests, their continued presence. Removal of C2C led to a gradual recovery of calcium influx capacity reaching 90% of pretreatment levels in 48 h while continued presence of C2C blocked recovery of calcium influx capacity (Fig. 2d). In contrast, SH-SY5Y cells did not recover calcium influx capacity with either the removal or the continued presence of latrunculin A. The reversibility of the effect of C2C treatment on calcium influx capacity indicates C2C is temporary in action and may undergo intracellular titration, degradation or enzymatic alteration^35,36^.

### Targeting specificity of neuronal subclass

**Figure 3:**
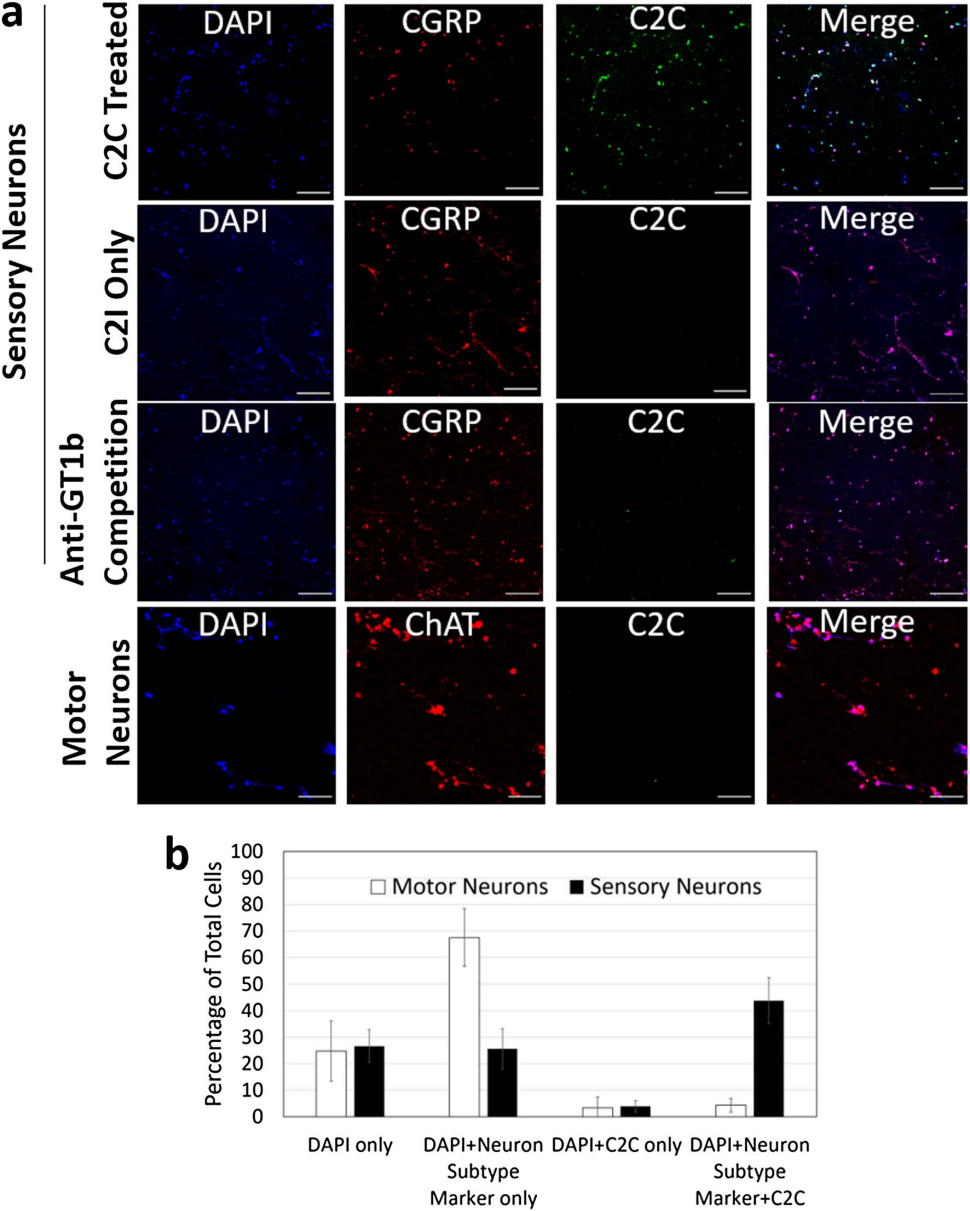
In vitro specificity of C2C. **a** Primary sensory and motor neurons were cultured from chicken embryos and treated with 120 nM of fluorescently derivatized C2C (green). Sensory neuron cultures were stained with sensory neuron marker anti-calcitonin gene related peptide (CGRP, red) and DAPI DNA stain (blue). Motor neuron cultures were stained with motor neuron marker anti-choline acetyltransferase (ChAT, red) and DAPI (blue). **b** Presence of C2C in neuron subtype positive cells was quantitated and compared between motor and sensory neurons. Cell counts were taken in eight fields of views in two biological replicates. Error bars represent standard deviation between all fields of view.

In prior studies C2C was shown to target immortal neuronal and nonneuronal cell lines in a GT1b-stimulated manner^19^. To assess its targeting specificity with primary neurons, sensory and motor chicken neuronal lines were established as described^25,37^. These cells were then probed with C2C prepared with fluorescently derivatized C2I, the G-actin ribosylase component of C2C. Neuronal subclass identity was determined using established neuronal markers. Sensory neurons were identified using antibodies against calcitonin gene-related peptide (CGRP)^38^. Motor neurons were identified using antibodies against choline acetyltransferase (ChAT)^39^. Nuclei and mitochondria were identified using DAPI^40^. Primary neuron cultures were established using dorsal root ganglia (DRGs) for sensory neuron cultures and spinal cord neurons for motor neuron cultures using at least three chicks for each experiment. The occurrence of GT1b was also demonstrated with chicken sensory neurons (Fig. S2). C2C colocalized with CGRP-positive cells but not with ChAT-positive motor cells (Fig. 3a). When sensory neurons were treated with the fluorescently labeled enzymatic component, C2I, but without the binding component, C2II-C1, there was no colocalization of C2I with CGRP. When sensory cells were pretreated with antibodies against the ganglioside GT1b, there was no colocalization of C2I with CGRP (Fig. 3a) and higher magnification images (Fig. S3). These data indicate that C2C preferentially targets primary sensory neurons and that C2I delivery is dependent on C2II-CI.

The magnitude of this specificity was determined by cell counting. Primary sensory and motor neuron cells were quantitated for the number of cells that stained positively for DAPI, for the neuron subtype marker, and for fluorescent C2I. Five fields of view were quantitated in two biological replicates for motor and sensory neurons to reach a minimum of 500 cells for each neuronal subclass treatment. Counted cells were placed into the following categories: positive for DAPI and negative for neuron subtype marker and C2C, positive for DAPI and neuron subtype marker and negative for C2C, positive for DAPI and C2C and negative for neuron subtype marker, and positive for DAPI, neuron subtype marker, and C2C (Fig. 3b). Consistent with prior reports, these primary neuron subclass enrichments contained a diversity of cells as reflected in the respective fractions for DAPI and different subtype markers^25,37,41,42^. Motor and sensory neuron cultures were positive for DAPI only in 24.8% and 26.6% of cells respectively^25,41,43^. Motor and sensory neuron cultures were positive for DAPI and C2C only at 3.4% and 3.9% of cells respectively. Motor and sensory neuron cultures were positive for DAPI and neuron subtype marker in 67.5% and 25.7% of cells respectively. Finally, motor and sensory neuron cultures were positive for DAPI, neuron subtype marker, and C2C in 4.3% and 43.8% of cells respectively.

### In vivo targeting of peripheral mouse sensory neurons and nerve fibers

**Figure 4:**
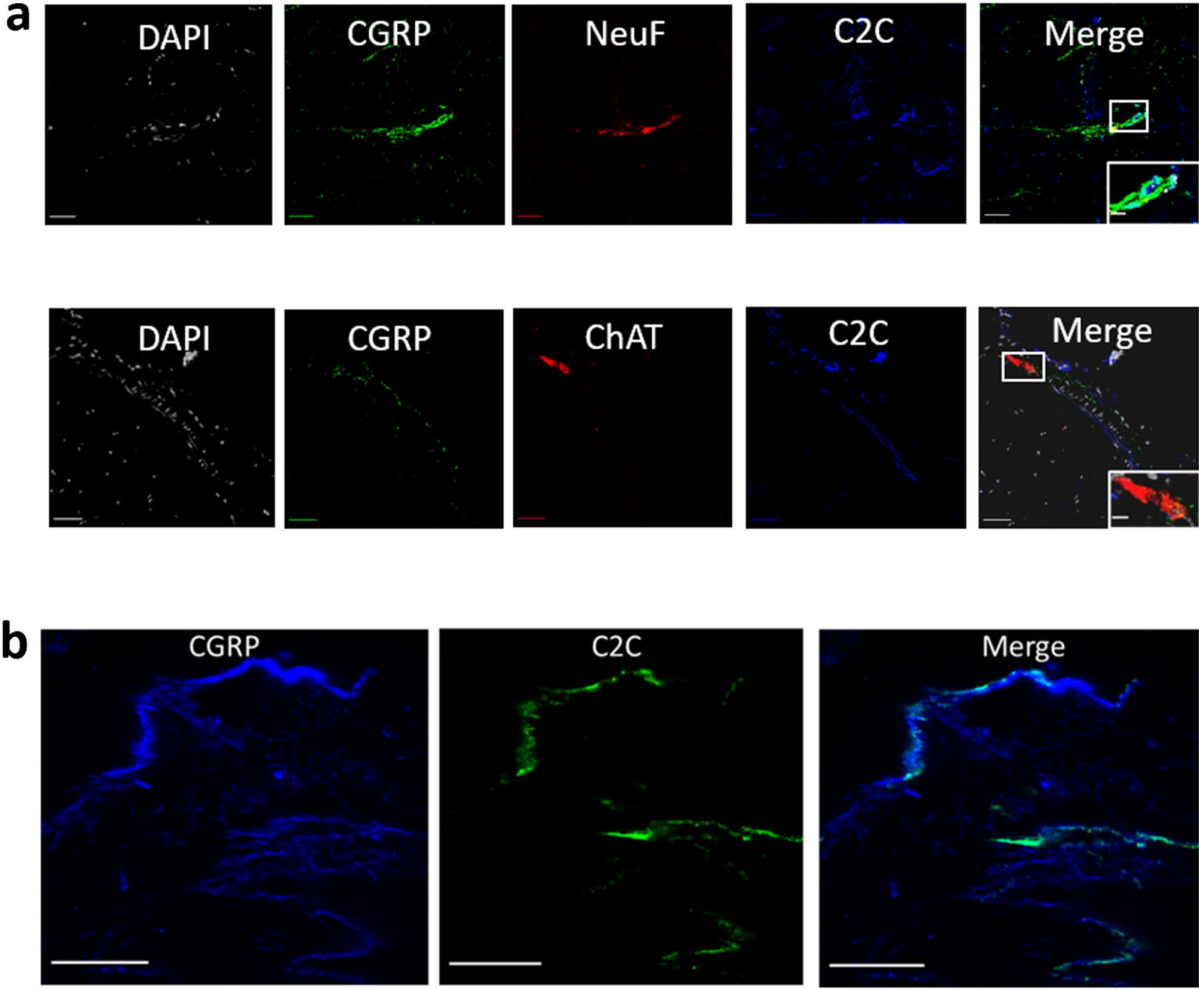
In vivo targeting of mouse sensory nerve fibers by C2C. Latissimus dorsi samples were obtained after subcutaneous administration of fluorescently derivatized C2C followed by treatment with antibody reporters. **a** Cryosections. Top row; DAPI DNA stain (white), sensory neuron marker anti-calcitonin gene related peptide (CGRP, green), general nerve fiber marker anti-neurofilament (NF, red), C2C (blue). Bottom row; as for top row but anti-choline acetyltransferase (ChAT) replaces NeuF (red). Scale bar is 20 µm, inset scale bar is 5 µm. **b** Whole mounts with 1.2 µm optical slices; anti-calcitonin gene related peptide (CGRP, blue), C2C (green). Scale bar is 30 µm.

To assess the specificity of C2C targeting in animals, its localization with peripheral neurons and nerve fibers was examined in mouse tissue using immunofluorescence microscopy. C2C was prepared using fluorescent C2I and exhibited identical potency as C2C prepared using non fluorescent C2I. It was then injected subcutaneously in the back of mice as described^44^. After 6 h, animals were euthanized and both fresh and fixed tissues from the latissimus dorsi muscle were removed, and either cryosectioned before imaging (Fig. 4a) or examined as whole mounts (Fig. 4b). Images shown are representative of at least three mice for all markers. Cell type identity was determined using fluorescently labeled antibodies specific for the sensory neuron marker, calcitonin gene-related peptide (CGRP)^38^^,^ the motor neuron marker, choline acetyltransferase (ChAT)^39^^,^ and Neurofilament (NeuF) a general neuronal marker along with DAPI for nuclear and mitochondrial DNA. C2I co-localized with nerve fibers detected by NeuF but only those that were CGRP-positive (Fig. 4a, top row). In this nerve fiber, fluorescent C2I was seen co-localizing with some sections that were CGRP-positive producing a cyan color that results from the combination of the green CGRP and the blue C2I (Fig. 4a, top row, inset). C2I did not colocalize with ChAT positive fibers (Fig. 4a, bottom row and inset). C2I also colocalized with CGRP-positive afferent sensory nerve fibers in 1.2 µm optical slices of whole mount tissues (Fig. 4b) where C2I is green and CGRP is blue. In addition, C2I (green) colocalized with CGRP-positive (blue) afferent nerve fiber in compiled optical slices of whole mount tissue (Fig S4). Fluorescent C2I (green) was also observed throughout the length of axons of sensory neurons (Fig. S5). This may indicate a propensity to undergo intracellular diffusion. While C2I colocalized with CGRP, this association occurred in only a subset of CGRP-positive locations. This is consistent with limited distribution of the GT1b receptor combined with intracellular diffusion of C2I. Overall, these results indicate C2C targets sensory nerve fibers not motor nerve fibers in vivo and are consistent with its apparent in vitro specificity targeting primary sensory not motor neurons.

### Effect of C2C on nociceptive pain in BALB/c mice

**Figure 5:**
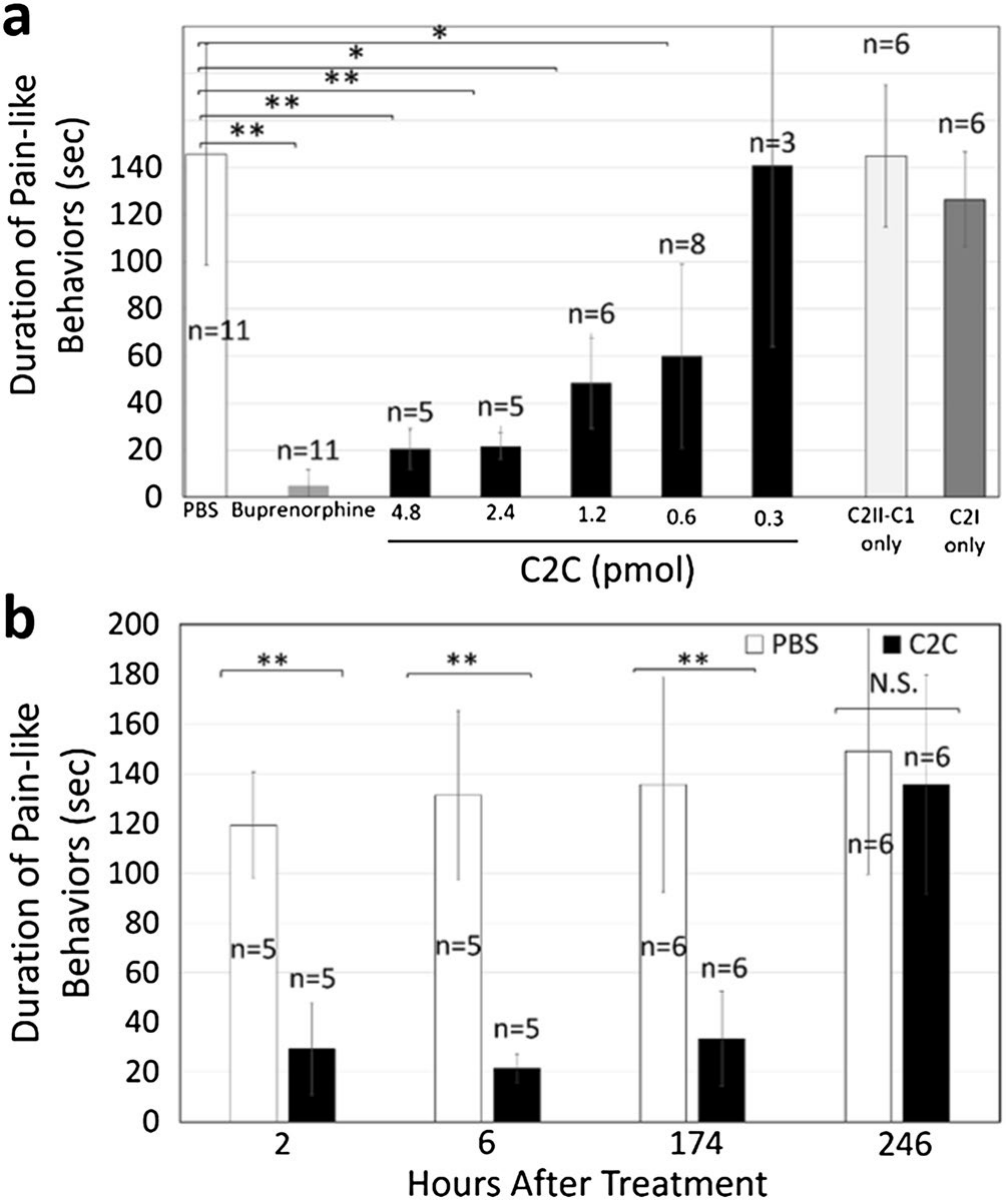
Effect of C2C on pain-like behaviors in BALB/c mice. The formalin nociceptive pain test was utilized to evaluate the dose and duration of responses to C2C administration in BALB/c mice. Shown are data for female mice ages 8–10 weeks. **a** Dose response of C2C (black bars) relative to buprenorphene (grey bar, 12.82 nmol), carrier (open bar, PBS), C2C subunits C2I and C2II-CI (light and dark grey respectively, 1.2 pmol). Numbers of animals used are indicated over each bar (n). **b** Duration of C2C effect. A single dose of C2C (2.4 pmol) was administered followed by formalin after 2 h, 6 h, 174 h (7.2 days), and 246 h (10.2 days). Statistical significance is indicated by *(p < 0.05), or **(p < 0.01).

Because C2C reduced calcium influx and preferentially targeted sensory neurons and nerve fibers in vitro and in vivo, it was predicted it would influence pain-like behaviors in an animal model for peripheral nociceptive pain. To test this possibility, the effect of C2C administration was examined in the inflammatory formalin test^45^. BALB/C female mice were treated either with C2C, each of the subunits (C2II-C1 and C2I) individually, and with controls by subcutaneous (SC) injection into the lateral, dorsal surface of the hind paw. The response to a range of C2C doses and the duration of the response to a single dose were examined. The total duration of all behaviors along with the total number and duration of each behavior were recorded over a 30-min window following an initial 5 min acute phase as described^45^. Statistical and power analyses of the experimental design and results used SAS 9.3 statistical software and G* power 3.1. Six h after administration, a C2C dose of 2.4 pmol blocked 90% of pain-like behaviors relative to the carrier only (PBS). The effect of C2C closely approximated the amount of reduction in pain-like behaviors observed with the opioid, buprenorphine, administered identically but at a higher dose 12.82 nmol (Fig. 5a). A two fold increase in C2C dose (4.8 pmol) conferred no additional benefit. Reduced amounts of C2C conferred correspondingly less reduction in pain-like behavior with a minimum effective dose of 0.6 pmol. Similarly, administration of either C2C protein subunits, C2II-CI or C21, produced no reduction in pain-like behavior. This indicates that delivery of C2I by C2II-C1 is a requirement for reduction in pain-like behavior by C2C. Pain-like behaviors were binned in 5-min increments and then summed for each treatment for dose response to distinguish between phase I and phase II response periods (Fig. S6). At all doses tested, the effect of C2C on pain-like behavior achieved a power of 0.8. C2C was equally effective in reducing pain-like behavior in both female and male animals using a dose of 2.4 pmol 6 h post treatment (Fig S7). No change in normal motor function was evident in all treated animals as indicated by the digit abduction test^46^ conducted on all tested animals. Similarly, no change was observed in any C2C treated animal in normal grooming, eating, other behaviors, or in weight gain during the period of evaluation.

Local anesthetics typically target ion channels thereby inhibiting formation or propagation of an action potential. They are uniformly short acting in duration largely because they diffuse away from the site of action and are subsequently taken up by non-neuronal tissues^47^. The longest acting form, liposomal bupivacaine, extends this duration to 3 days^48^. Because C2C is a protein and cannot readily diffuse away from the site of action and also acts intracellularly through import by receptor mediated endocytosis, it was of interest to measure its duration of action. This was done using the formalin mouse pain model by varying the period of incubation following a single dose (2.2 pmol) of C2C before formalin treatment (Fig. 5b). Pain-like behaviors were also binned in 5-min increments and then summed for each treatment for duration (Fig. S8). Incubation periods of 2 h, 6 h, 174 h (7.2 days) and 246 h (10.2  days) were tested. Inhibition of pain-like behavior was evident within 2 h of treatment and lasted throughout the 174 h (7.25 day) period. There was a gradual decrease in the amount of reduction of pain behavior after this period, until there was no reduction in pain-like behaviors at 246 h (10.2 days). Again, statistical and power analysis of these data used SAS 9.3 and G* power 3.1 and achieved a power of 0.8. Similarly, no change in normal motor function was evident as indicated by the digit abduction test^46^ and no change was observed in grooming, eating, other behaviors, or weight gain during the period of evaluation.

## Discussion

C2C is an engineered protein designed to reduce pain signaling through delivery of an actin remodeling enzyme to peripheral sensory neurons by GT1b receptor targeting. C2C was shown to inhibit pain-like behaviors in a mouse formalin pain model in a manner similar to local anesthetics but with novel features. While C2C imparted pain relief slowly, having full activity 2 h post administration, this effect continued for one week. This exceeds the duration of action of local anesthetics including those designed for extended release^49,50^.

In vitro, C2C reduces calcium influx in primary chick sensory neurons and immortal human neuroblastoma cells. Anesthetics, which are uniformly small molecules such as lidocaine, also reduce ion flux and thereby limit formation or propagation of an action potential^51^. In vivo, these effects reduce neuronal pain signaling. Despite its molecular complexity, C2C exhibits a greater molar potency than local anesthetics^52^. C2C had a maximum effective dose of 2.4 pmol in the animal pain test model. This effect plateaued at higher doses suggesting occurrence of target saturation, but it remains to be seen if this results from titration of receptor binding or limited recycling, G-actin ribosylation or calcium channel disruption. Additional in vitro studies using cultured primary chick neurons and fluorescence microscopy demonstrated that C2C is specific for sensory neurons and not motor neurons. This targeting enables preferential intracellular delivery of the G-actin ribosylase and arises from specificity of the C2C oligomeric subunit, C2II-C1, for the GT1b ganglioside receptor^19^. Unlike other clostridial proteins such as Botox, GT1b appears to be necessary and sufficient for C2C binding and is not co-dependent on protein receptors^22^. Multiple types of calcium channels are known to be dependent on actin polymerization in neuronal cells^53^. The reduction of calcium influx does result in a reduction of action potential initiation^54^. Actin dependence of calcium influx is thought to be through uptake of calcium because of recruitment done by calcium channel trafficking, that has been shown to be inhibited by other actin polymerization inhibitors^55^. This could be a similar model for the action of C2C in inhibiting calcium influx. Such specificity provides a mechanism to perform actin remodeling in only a subset of neurons using an intracellular mechanism.

Histologic studies of cryosectioned and whole tissue mounts examined using confocal laser microscopy of fluorescent C2C and various reporters of neuron subclasses, demonstrated specificity of C2C towards sensory neurons and sensory fibers in mouse peripheral tissue. Interestingly C2C was well distributed along sensory neuron axons that innervated muscle tissue. This may indicate the C2I protein undergoes intracellular diffusion as has been observed with other neuron targeting proteins^56^ and it suggests that C2C could influence calcium channel activity at locations distal to its site of injection. Alternatively, the apparent distribution of C2C is also consistent with GT1b receptor location along sensory neuron axons in mouse tissue. Most importantly, C2C localized to afferent sensory nerve fibers but did not colocalize with efferent motor nerve fibers. GT1b is present on > 90% of peripheral sensory neurons in mice^57^ and to some extent on motor neurons but to a lesser extent^57^. It also has been reported that there is a higher amount of GT1b found on sensory neurons than motor neurons in tissue collected from human cadavers^58^. In addition, it has been shown that injuries to the peripheral nervous system results in increased expression of ganglioside GT1b^59^. It may be possible that C2C binds some motor neurons but a combination of reduced uptake and insufficient GT1b-positive motor neurons leads to a difference in C2C activity in different neuron subtypes. Additional mechanisms are also plausible. It is known that GT1b is present on most mammal species that have been tested for its presence^60–62^. This specificity was consistent with the lack of an apparent effect on motor function in animal tests.

C2C disrupts calcium influx in primary sensory neurons and immortal SH-Sy5Y cells in a dose dependent manner. The utility of the SH-SY5Y response could allow for more robust and quantitative studies on the effect of C2C on calcium channel inhibition. For example, C2C removal following primary sensory neuron treatment in vitro, restored calcium influx capacity. The mechanism underlying this restoration could be examined for kinetic and dose response relationships. This reversibility also is consistent with the behavioral data indicating that signal dose administration produces a transitory effect ending one week post administration.

## Materials and methods

### Preparation of C2C

C2C was prepared as described previously^19^. Briefly, plasmid encoded expression constructs for C2I and C2II-C1 were expressed in *E. coli* BL21. Cell lines were grown in LB medium, with ampicillin (100 µg/mL) at 37 °C, and induced at an optical density of ~ 0.6 at 600 nm wavelength with 0.5 mM IPTG. A french pressure cell was used to lyse cell paste with 10,000 psi pressure. Glutathione resin (Genscript) was used for affinity purification. GST fusion tags were removed using thrombin (Thermo Fisher). C2II-C1 was further activated using trypsin by incubation at 37 °C for 30 min at a 1:5 enzyme to substrate ratio as previously described^63^.

### Cell culture

The SH-SY5Y cell line was used for some ion flux analysis. It is an immortal human neuron-like cell line derived from glioblastoma^64^. Cells were cultured at 37 °C with 5% CO_2_, as described^65^. Chicken sensory neurons were cultured from chicken embryo dorsal root ganglia as described^25^ using embryos at Hamburger stages 31–36^66^. Chicken sensory neurons were cultured in Ham’s F12 media. Nerve growth factor (1.25 ng/mL) and Neurotrophin 3 (1.25 ng/mL) were added for neuronal differentiation. Chicken motor neurons were cultured from chicken embryo spinal cords as described^37^ using embryos at Hamburger stages 29–34^66^.

### Actin fluorescence assays

Primary chicken sensory neurons and GT1b containing SH-SY5Y cells were tested for staining using Alex Fluor 488 phalloidin (Invitrogen) with modification^67^. Sensory neurons were cultured on acid washed, round coverslips in black sided 24 well plates (Corning) or on glass coverslips. Primary cells were allowed to incubate at 37 °C for 48 h in hormone containing media. Following incubation, cells were treated with latrunculin A (Sigma) and C2C at the doses indicated for 2 h. After incubation, the medium was removed from the wells and cells were washed with PBS pH 7.2. Cells were fixed with 4% paraformaldehyde for 30 min at room temperature. Cells were washed 2 times with PBS then permeabilized with 0.5% (v/v) Triton X-100 for 10 min, then washed with PBS 2 times. Cells were stained with phalloidin-488 for 30 min at room temperature. Microscopy used a Nikon A1R confocal microscope. Imaging software was Nikon Instruments (NIS). A fluorescent plate reader was used to measure 30–40,000 cells per well and an average of three wells for each dose.

### Immunocytochemistry

Coverslips containing primary sensory and motor neurons were probed with C2C prepared using fluorescently derivatized C2I. Derivatization used an Alexa fluor micro labelling kit as described by the supplier (Thermo Fisher). Cells were incubated with fluorescent C2C for 2 h at 37 °C in a CO_2_ incubator. Cells were fixed with a fresh 4% (w/v) paraformaldehyde solution at room temperature for 15 min. Coverslips were blocked before antibody addition with 5% (v/v) normal goat serum (Sigma) for 1 h at room temperature. Sensory neurons were identified using antibodies against calcitonin gene-related peptide (CGRP, Abcam, Cat # ab26001) with a 1:300 dilution^38^. Motor neurons were identified using antibodies against choline acetyltransferase (ChAT, Abcam, Cat # ab18736) with a 1:300 dilution^39^. Coverslips were then mounted on slides using fluorogel mounting solution (Thermo Fisher). A Nikon A1R confocal microscope was used to examine cultured cells. Imaging software was Nikon Instruments (NIS) and Image J.

### Whole-mount and cryosection preparations

C2C was prepared using fluorescently derivatized C2I then injected subcutaneously into the latissimus dorsi of 8–10 week old female BALB/c mice. Following C2C administration and 6 h subsequent incubation, mice were euthanized and perfused with 30 mL of 0.9% saline solution, followed by 30 mL of 4% paraformaldehyde solution. Tissue preparations were dissected from the latissimus dorsi and prepared as cryosections and whole-mount samples. Tissue was fixed with 4% paraformaldehyde diluted in phosphate buffered-saline at 25 °C for 1 h. In addition, whole-mount tissue samples were fixed with 4% paraformaldehyde overnight at 4 °C. Following fixation whole-mount samples were washed with phosphate-buffered saline, pH 7.2 (PBS) twice to remove fixative. Samples of latissimus dorsi were frozen for cryosectioning in OCT medium (Thermo Fisher) on dry ice. Frozen tissue was sectioned using a cryostat at a width of 10–20 µm. Sections were mounted onto polyethyleneimine coated slides. Cryosections were blocked with 5% (v/v) normal goat serum (Sigma) and washed with Tween 20 (0.1% v/v) in PBS three times for 10 min each. Whole-mount tissue was permeabilized with 0.05% Triton X-100 TBS solution for 3 h at 4 °C. Whole-mount tissue was blocked with 5% (v/v) normal goat serum for 3 h at 4 °C. Primary and secondary antibody incubations were performed for 1 h for cryosections and 16 h for whole-mount preparations. Sensory neurons were identified using antibodies against calcitonin gene-related peptide (CGRP, Abcam, Cat # ab26001) with a 1:300 dilution^38^. Motor neurons were identified using antibodies against choline acetyltransferase (ChAT, Abcam, Cat # ab18736) with a 1:300 dilution^39^. Neurofilament (NeuF, Abcam, Cat # ab204893) was used to identify general nerve fibers. Nuclei and mitochondria were identified using DAPI (Sigma)^40^. Coverslips were then mounted on slides using fluorogel mounting solution (Thermo Fisher). A Nikon A1R confocal microscope was used to examine cryosections and whole-mount tissue. Whole-mount images were taken with a z-series of 60–200 um depth, with individual images of 1–2 µm optical slices. Imaging software was Nikon Instruments (NIS).

### Ion influx assays

Primary chicken sensory neurons and non-stimulated SH-SY5Y neuroblastoma cells were tested for calcium influx or sodium influx using fluor-4 (Thermo Fisher) or SBFI sodium indicator (Thermo Fisher) respectfully as described^65,68^ with modification. Sensory neurons were cultured on acid washed, round coverslips in black sided 24 well plates (Corning). Cells were allowed to incubate at 37 °C for 48 h in hormone containing media. Following incubation, cells were treated with latrunculin A (3 µM, Sigma) and C2C at the doses indicated for 2 h. After incubation, the medium was removed from the wells and cells were washed with PBS pH 7.2. Fluo-4 calcium indicator in kit assay buffer containing 1.26 mM calcium or SBFI sodium indicator in an assay buffer containing 1.1 mM sodium was added into each well and incubated for 30 min at 37 °C, followed by a 30 min incubation at room temperature. A fluorescent plate reader was used to measure the flux of ion indicators using 30–40,000 cells per well and an average of three wells for each indicator.

### Animal behavior tests

Animal experimental procedures were performed in accordance with the National Institutes of Health Guide for Care and Use of Laboratory animals. Experimental procedures involving animals were approved by the Institutional Animal Care & Use Committee of University of Nebraska-Lincoln. The animal behavior test for acute pain was done using the formalin test^45,69^. Mice were anesthetized using isoflurane prior to all injections. In dose response experiments and after anesthesia, mice were subcutaneously injected with 20 µL of sterile filtered C2C in the amounts indicated ranging from 0.6 to 4.8 nmol into the dorsal, lateral surface of the left hind paw. In duration studies, mice were anesthetized with isoflurane, then subcutaneously injected with 2.4 pmol of C2C and incubated prior to formalin challenge in times ranging from 2, to 174 h (7.25 days). In all procedures, mice were treated with PBS as a negative pain relief injection control or with 12.82 nmol buprenorphine as a positive pain relief control. Additional mice were treated with 1.2 pmol of each individual component of C2C, C2I and C2II-C1, to test if they produced any reduction in pain-like behavior. Following initial treatments, mice were placed in housing cages for 6 h. Following the incubation period, mice were moved to viewing containers and allowed to equilibrate to the new environment for 15 min. Mice were then anesthetized and subcutaneously injected with 20 µL of 5% (v/v) sterile filtered formalin into the dorsal, medial surface of the left hind paw. Mice were then moved back to the viewing containers and a video recorder was used to monitor behavior of mice for 30 min. Pain-like behavior was recorded as “hind paw licking behavior” indicated as licking of the dorsal surface of the left hind paw. Video recordings were then evaluated during the 30 min recording by counting the total duration of behavior, the number of behaviors, and the duration of each individual behavior. Blinding was performed in all animal behavior studies. A third party not present at the time of animal treatments recorded the pain-like behaviors in each video recording. Preliminary animal behavior studies used the formalin test to obtain pilot data for a power analysis necessary to determine the number of experimental animals needed to achieve statistical significance. The formalin test^45,69^ was used at 11 different C2C duration time-points and 5 different doses.

### Statistics

Formalin test statistical analyses were completed using the SAS 9.3 statistical software and G* power 3.1 The pilot data was treated as a factorial split plot, divided into 3 different levels of variables: (i) duration or dosage, (ii) treatment (C2C, untreated, buprenorphine control), and (iii) formalin test phase (phase 1 & 2). For the pilot data, a gamma distribution was selected based on the student residuals test. The PROC GLIMMIX procedure and LSMEANS were used to produce standard error means. The LSMEANS and standard error means were utilized to determine an effect size for each duration and dosage experiment. The effect size for each duration and dosage was used to determine the probability of correctly finding a difference within a given phase and duration or dosage. Using the effect sizes, and a cutoff of 80% power, and an alpha of 0.05, a power analysis was performed using G* power.

## Supplementary information

Supplementary information.
