# Supplementary material for: Intracellular G-actin targeting of peripheral sensory neurons by the multifunctional engineered protein C2C confers relief from inflammatory pain
Source: Sci Rep. 2020 Jul 30;10:12789. doi: 10.1038/s41598-020-69612-9 (PMC7393082; doi:10.1038/s41598-020-69612-9)
Supplement: Supplementary file 1 — Supplementary information. [file 41598_2020_69612_MOESM1_ESM.pptx]

## Slide 1
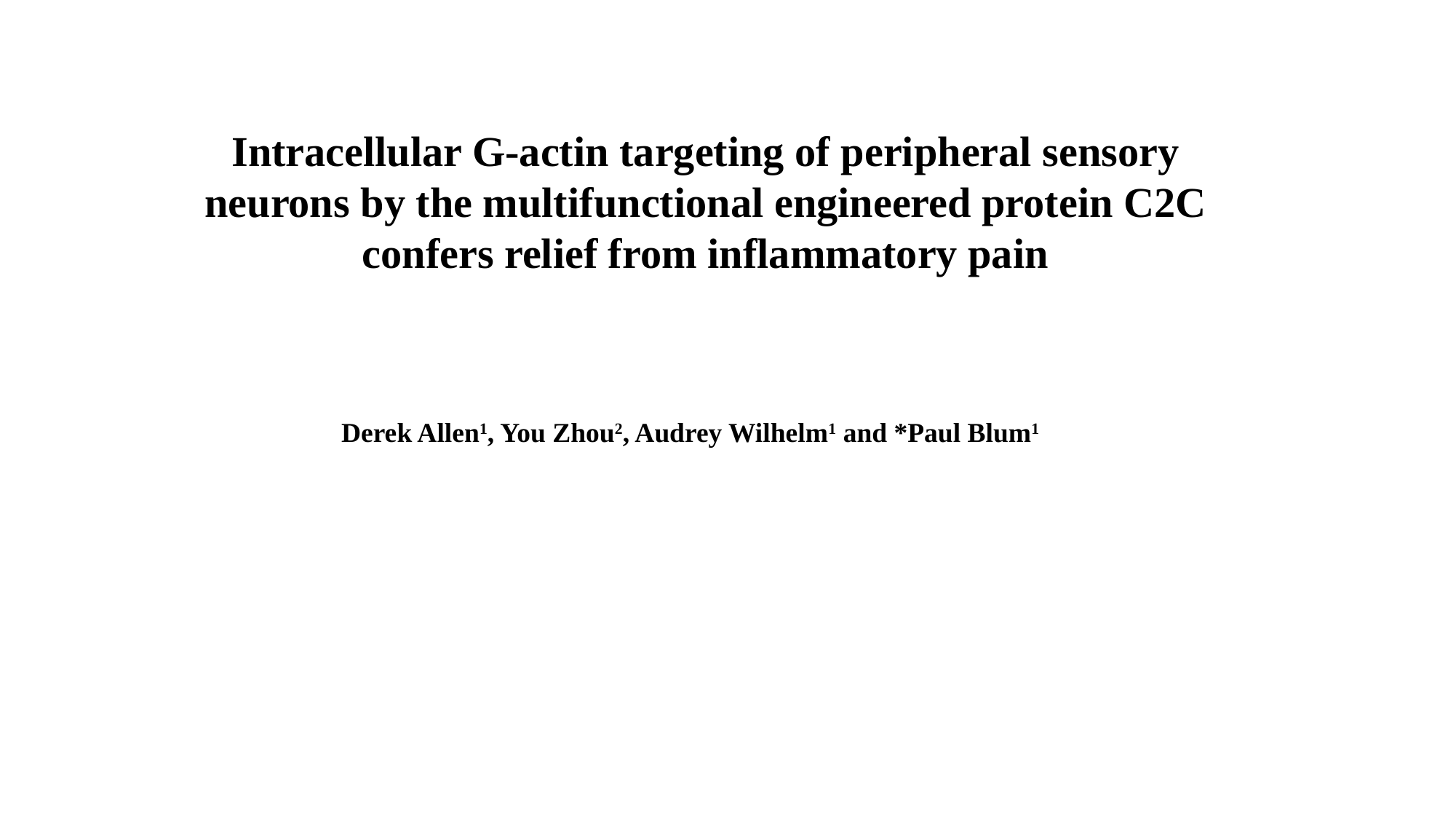

Intracellular G-actin targeting of peripheral sensory neurons by the multifunctional engineered protein C2C confers relief from inflammatory pain
Derek Allen1, You Zhou2, Audrey Wilhelm1 and *Paul Blum1

## Slide 2
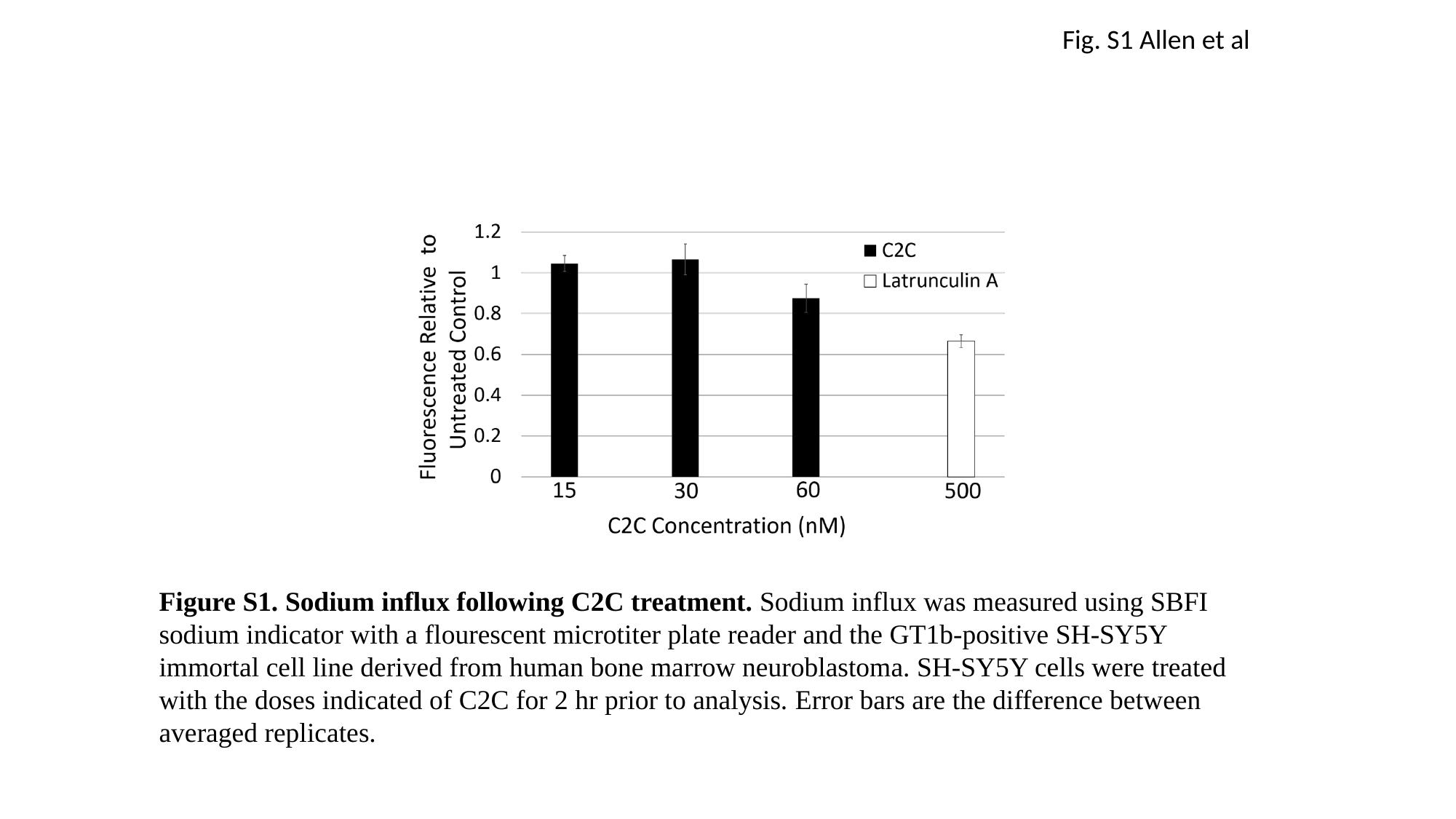

Fig. S1 Allen et al
Figure S1. Sodium influx following C2C treatment. Sodium influx was measured using SBFI sodium indicator with a flourescent microtiter plate reader and the GT1b-positive SH-SY5Y immortal cell line derived from human bone marrow neuroblastoma. SH-SY5Y cells were treated with the doses indicated of C2C for 2 hr prior to analysis. Error bars are the difference between averaged replicates.

## Slide 3
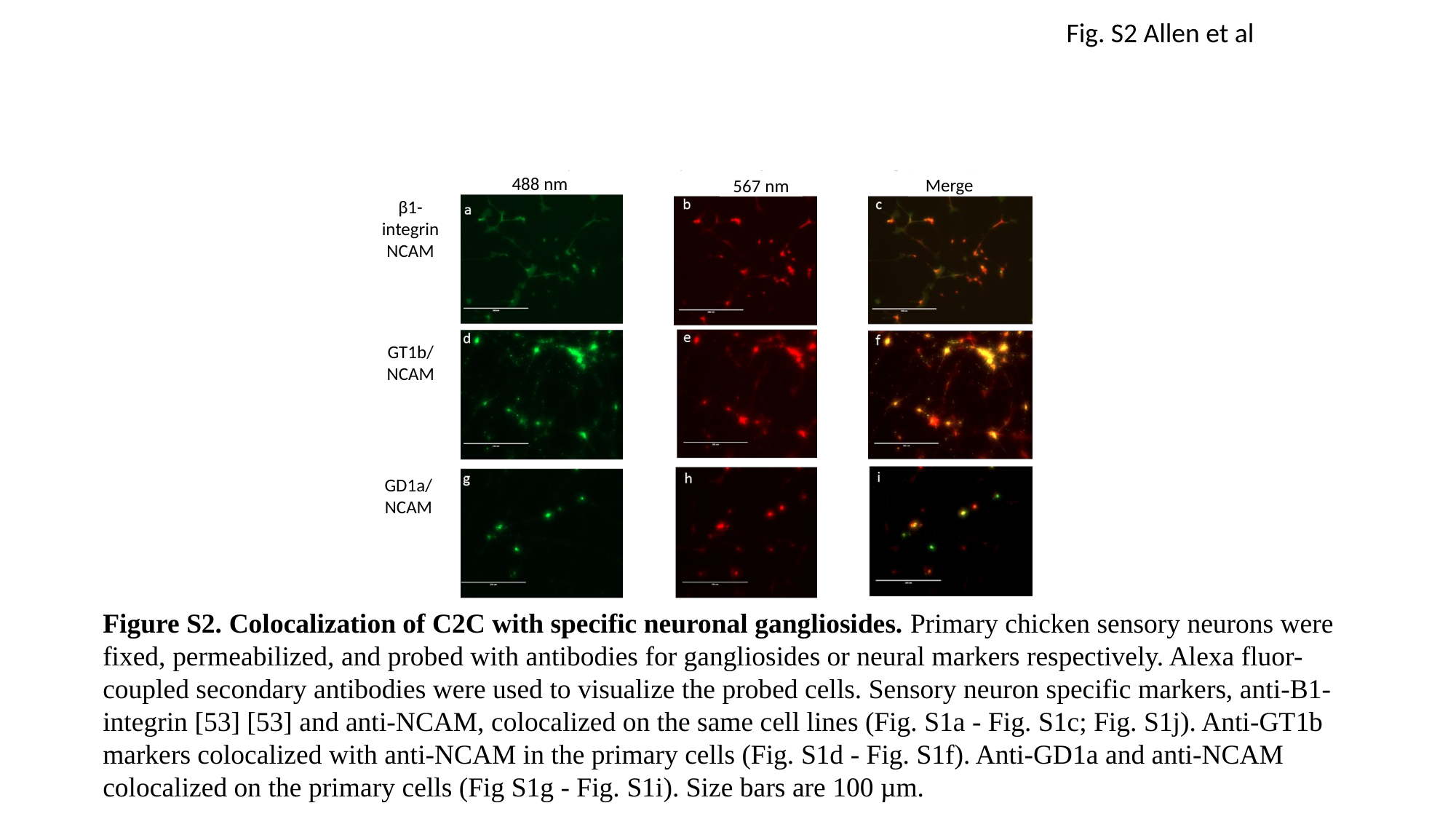

Fig. S2 Allen et al
488 nm
Merge
567 nm
β1-integrin NCAM
GT1b/ NCAM
GD1a/ NCAM
Figure S2. Colocalization of C2C with specific neuronal gangliosides. Primary chicken sensory neurons were fixed, permeabilized, and probed with antibodies for gangliosides or neural markers respectively. Alexa fluor-coupled secondary antibodies were used to visualize the probed cells. Sensory neuron specific markers, anti-B1-integrin [53] [53] and anti-NCAM, colocalized on the same cell lines (Fig. S1a - Fig. S1c; Fig. S1j). Anti-GT1b markers colocalized with anti-NCAM in the primary cells (Fig. S1d - Fig. S1f). Anti-GD1a and anti-NCAM colocalized on the primary cells (Fig S1g - Fig. S1i). Size bars are 100 µm.

## Slide 4
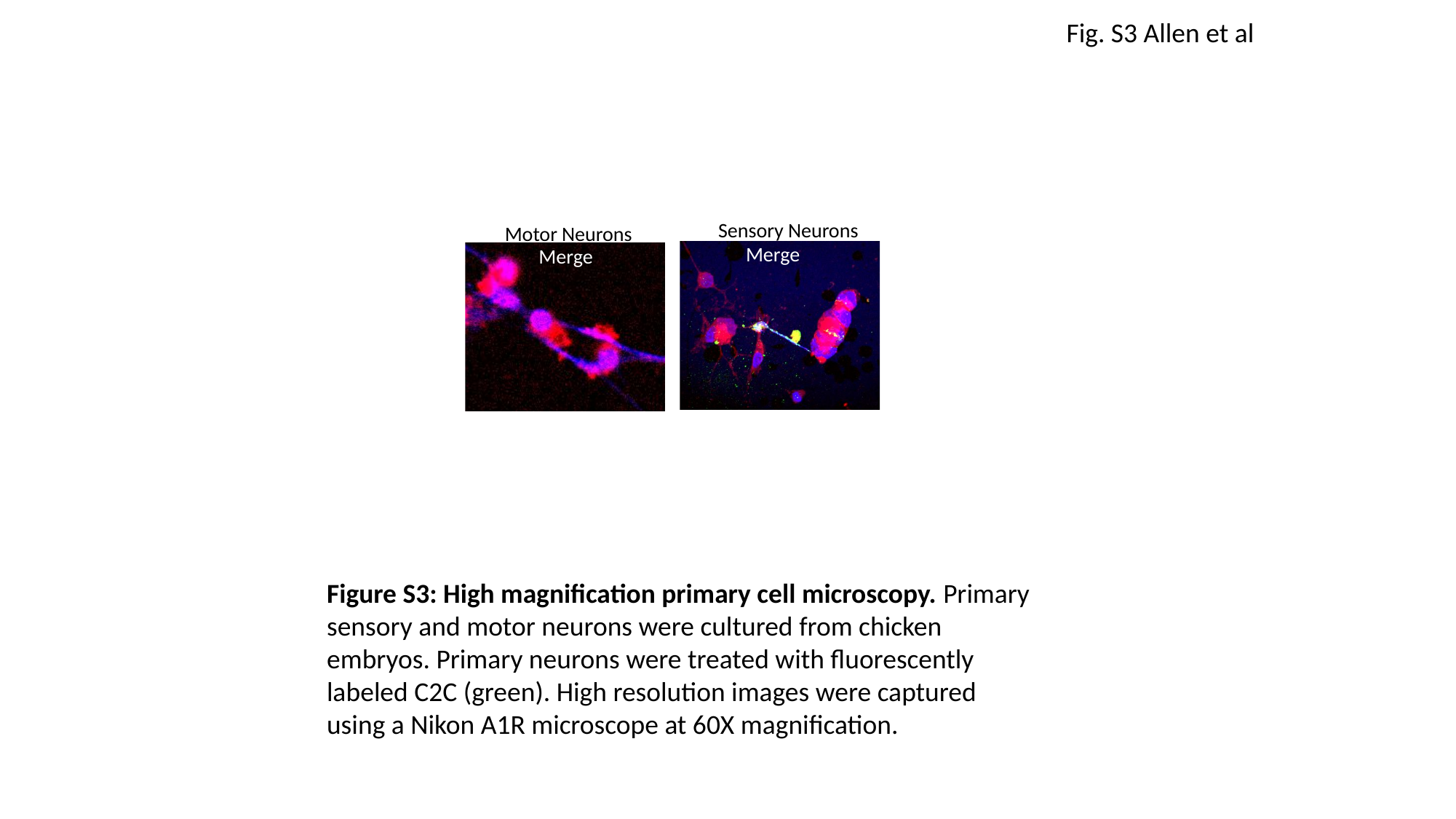

Fig. S3 Allen et al
Sensory Neurons
Motor Neurons
Merge
Merge
Figure S3: High magnification primary cell microscopy. Primary sensory and motor neurons were cultured from chicken embryos. Primary neurons were treated with fluorescently labeled C2C (green). High resolution images were captured using a Nikon A1R microscope at 60X magnification.

## Slide 5
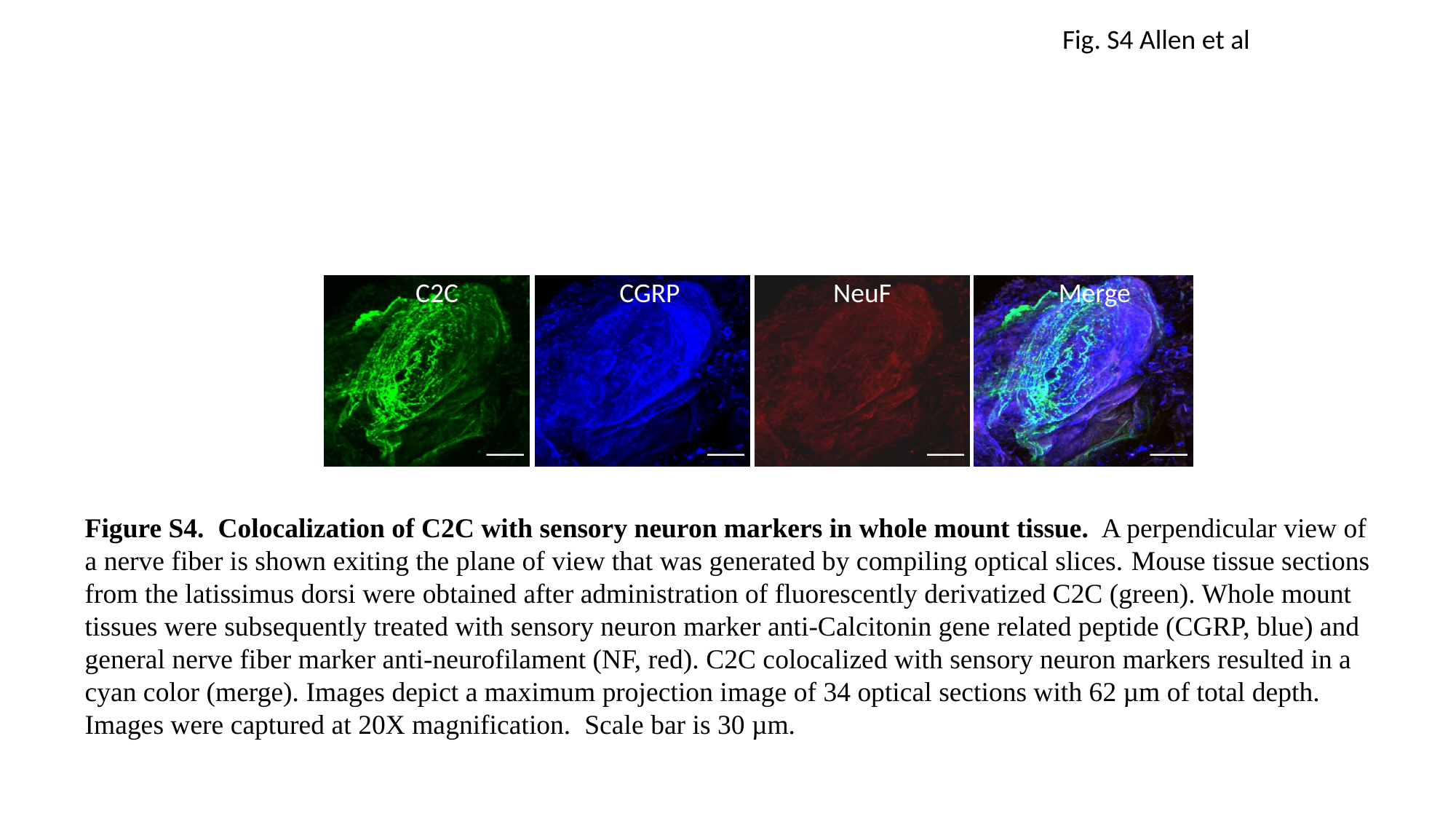

Fig. S4 Allen et al
C2C
CGRP
NeuF
Merge
Figure S4. Colocalization of C2C with sensory neuron markers in whole mount tissue. A perpendicular view of a nerve fiber is shown exiting the plane of view that was generated by compiling optical slices. Mouse tissue sections from the latissimus dorsi were obtained after administration of fluorescently derivatized C2C (green). Whole mount tissues were subsequently treated with sensory neuron marker anti-Calcitonin gene related peptide (CGRP, blue) and general nerve fiber marker anti-neurofilament (NF, red). C2C colocalized with sensory neuron markers resulted in a cyan color (merge). Images depict a maximum projection image of 34 optical sections with 62 µm of total depth. Images were captured at 20X magnification. Scale bar is 30 µm.

## Slide 6
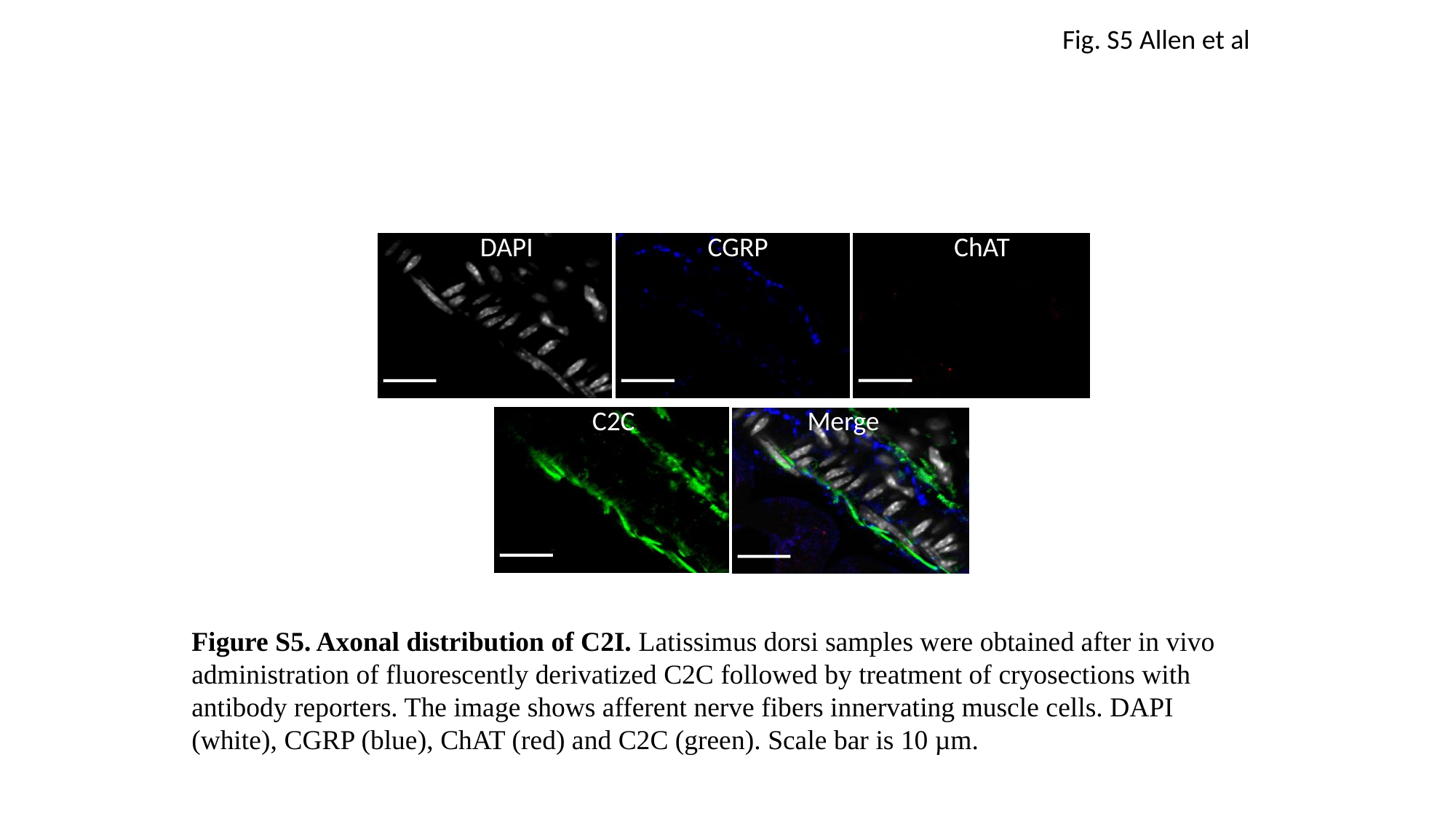

Fig. S5 Allen et al
DAPI
CGRP
ChAT
C2C
Merge
Figure S5. Axonal distribution of C2I. Latissimus dorsi samples were obtained after in vivo administration of fluorescently derivatized C2C followed by treatment of cryosections with antibody reporters. The image shows afferent nerve fibers innervating muscle cells. DAPI (white), CGRP (blue), ChAT (red) and C2C (green). Scale bar is 10 µm.

## Slide 7
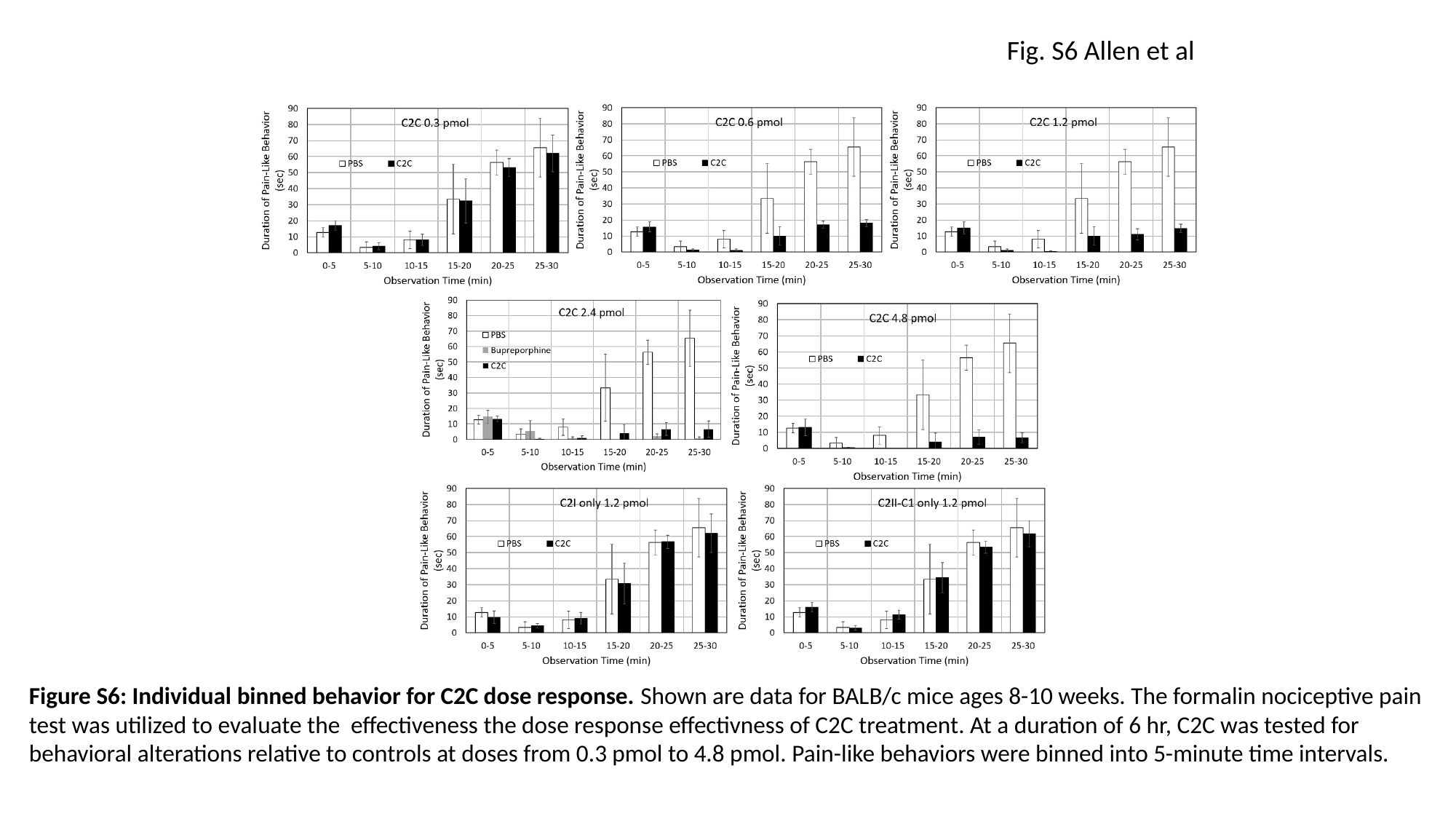

Fig. S6 Allen et al
Figure S6: Individual binned behavior for C2C dose response. Shown are data for BALB/c mice ages 8-10 weeks. The formalin nociceptive pain test was utilized to evaluate the effectiveness the dose response effectivness of C2C treatment. At a duration of 6 hr, C2C was tested for behavioral alterations relative to controls at doses from 0.3 pmol to 4.8 pmol. Pain-like behaviors were binned into 5-minute time intervals.

## Slide 8
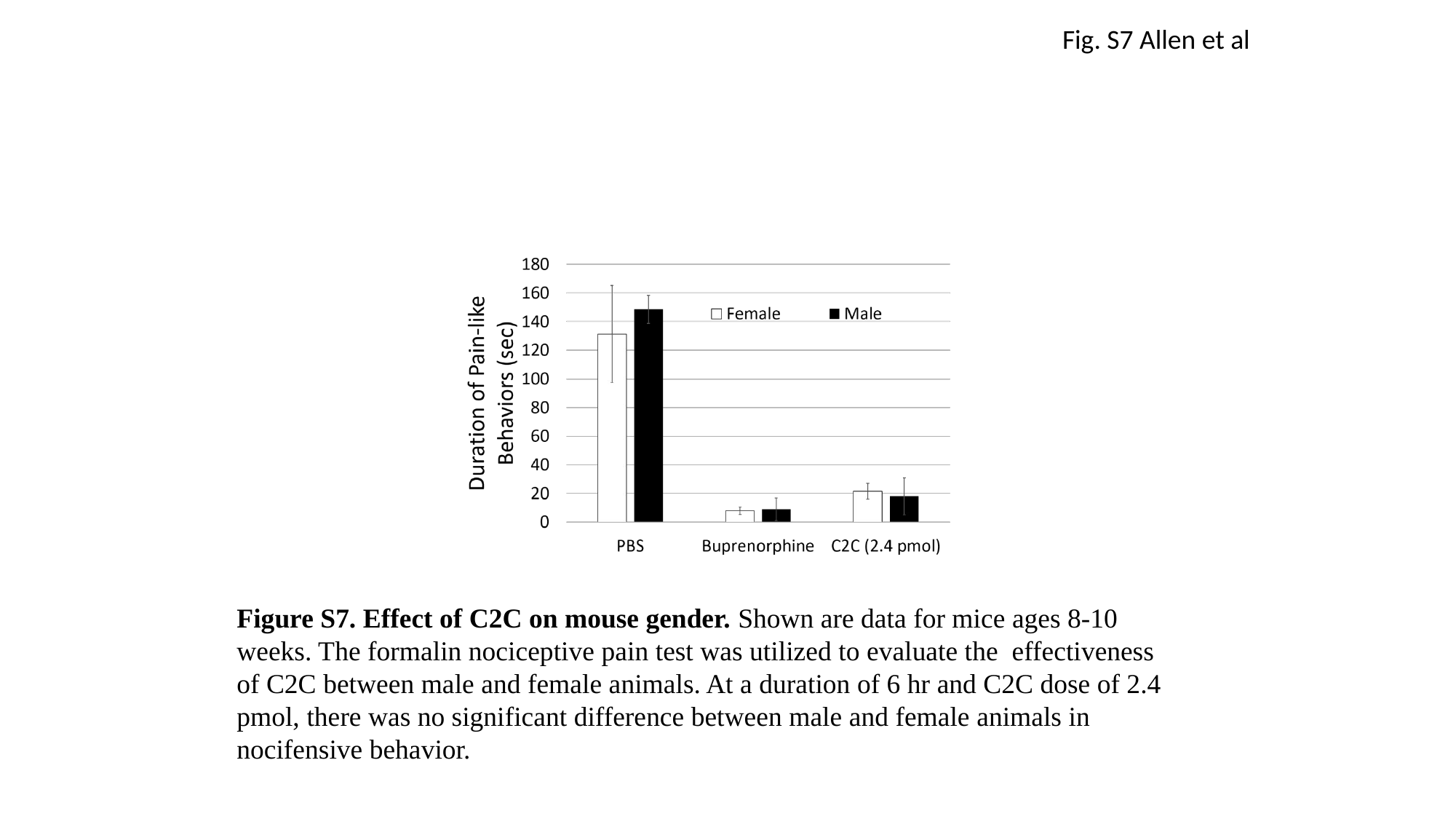

Fig. S7 Allen et al
Figure S7. Effect of C2C on mouse gender. Shown are data for mice ages 8-10 weeks. The formalin nociceptive pain test was utilized to evaluate the effectiveness of C2C between male and female animals. At a duration of 6 hr and C2C dose of 2.4 pmol, there was no significant difference between male and female animals in nocifensive behavior.

## Slide 9
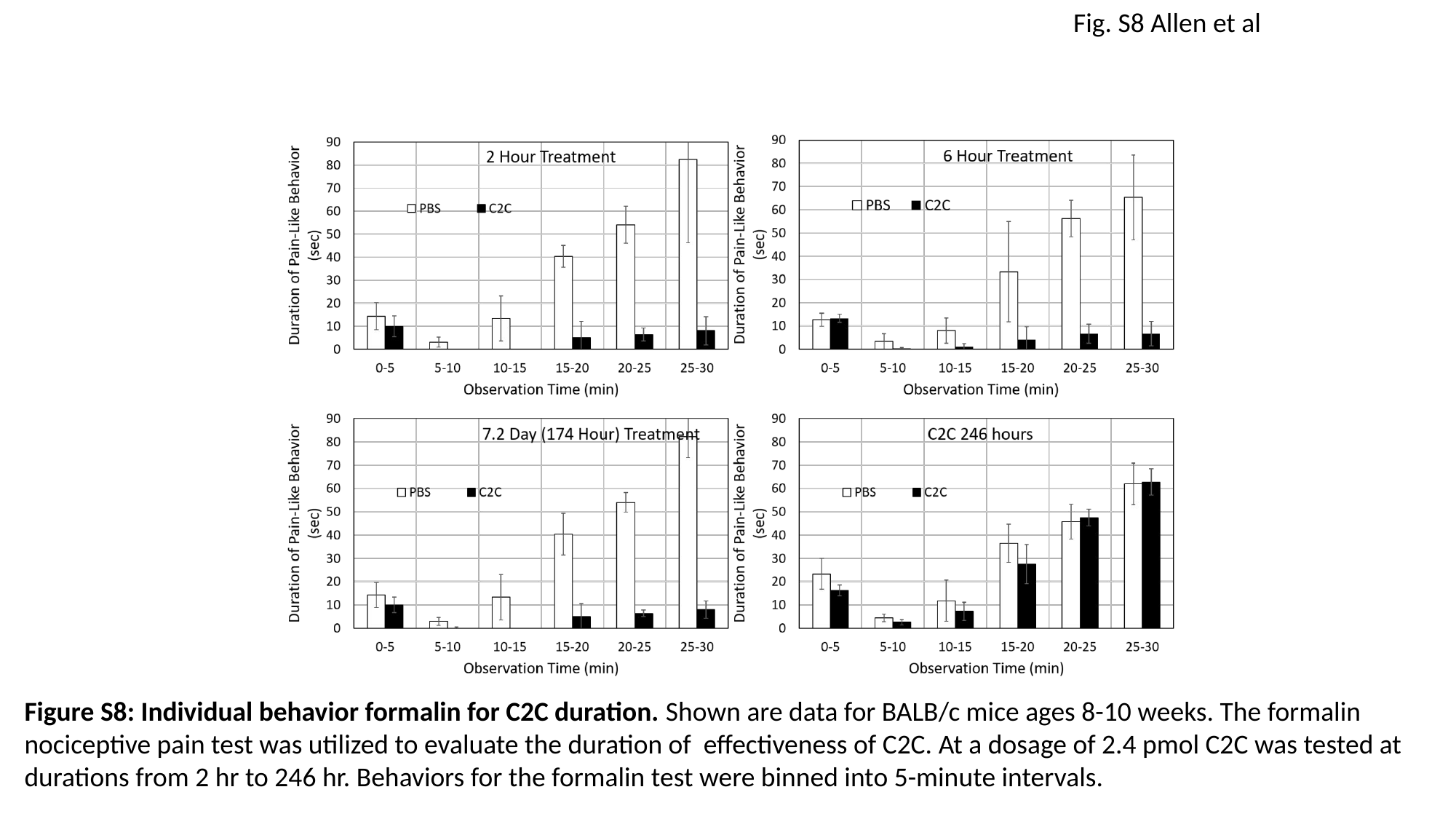

Fig. S8 Allen et al
Figure S8: Individual behavior formalin for C2C duration. Shown are data for BALB/c mice ages 8-10 weeks. The formalin nociceptive pain test was utilized to evaluate the duration of effectiveness of C2C. At a dosage of 2.4 pmol C2C was tested at durations from 2 hr to 246 hr. Behaviors for the formalin test were binned into 5-minute intervals.
